# Functional status, social support, and anxiety among postnatal women of Eastern India

**DOI:** 10.1016/j.eurox.2023.100238

**Published:** 2023-09-06

**Authors:** Nabanita Chandra, Moonjelly Vijayan Smitha

**Affiliations:** Obstetrical and Gynecological Nursing, College of Nursing, AIIMS Bhubaneswar, Odisha, India

**Keywords:** Daily living activities, Perceived social support, Family assistance, Spouse, Psychological adaptation, Puerperium

## Abstract

**Introduction:**

Though becoming a mother is a joyous experience, the postpartum time can be difficult and stressful for women as they deal with significant physical alterations and adjustments to their daily routines. However, very few studies have focused on the functional well-being of the woman after childbirth. This study aims to find the level of functional status, social support, and anxiety among women attending immunization clinics.

**Methods:**

A descriptive cross-sectional research design was adopted to recruit 220 women in this study from two immunization clinic centers in Bhubaneswar, Odisha. Data were collected using a self-reported sociodemographic profile, functional level scale after childbirth, modified multidimensional scale of perceived social support, and postnatal anxiety scale. Descriptive and inferential statistical tests were used for data analysis, including mean, percentage, and Fisher exact.

**Results:**

59.5 % of women returned to a moderate level of functional status after six weeks postpartum. The majority of women, 98.6 % and 83.6 %, reported high levels of return to personal care and baby care, respectively, whereas 34.7 % had moderate levels of return to home activities and 90 % had low levels of return to community and social tasks. Also, 70 % of women had high perceived social support, and 87.7 % had no anxiety. In this study, normal delivery women had better functional status than their cesarean delivery counterparts. Moreover, functional status was significantly associated with anxiety at six postpartum weeks.

**Conclusion:**

After six weeks of childbirth, most women only partially resumed their pre-pregnancy functional state. So, much more time, rest, and support from family members were needed to recover to a fully functional level. Nurses, midwives, and the family members of women should be aware of the critical role that social support plays in enhancing a woman's functional and psychological status during the postpartum period.

## 1 Introduction

In mother-child health services, postpartum care has a significant role. Generally, women gain more from prenatal care services than postpartum care [1]. Historically, the physiologic alterations and healing of the reproductive organs have been prioritized in assessments of postpartum recovery. The postpartum period's social and psychological components, which affect recovery in various ways, have received very little attention [1], [2], [3].

According to WHO, "the Postnatal period begins immediately after childbirth and extends to 6 weeks" [4]. The journey of becoming a mother during the postpartum time is difficult and stressful for the women since they must adjust to the increased responsibility of motherhood and significant physical changes [5].

Functional status, which encompasses the several dimensions of health, such as individual, biological, and social, characterizes a person's capacity to execute an activity or task in the environment in which they live [4]. In nursing research, functional status is a crucial term. To help women improve their health and the health of their families, nurses must re-examine the mother's role functioning [6]. "The readiness of a woman to resume her preferred degree of baby care, self-care activities, domestic activities, and occupational, social, and communal activities is referred to as functional status, a multidimensional phenomenon." [1], [5], [6], [7], [8]. In the Indian context, postpartum women are not permitted to go outside their homes for 40–42 days after giving birth [9], [10], [11], [12]. as it is thought to be a period of increased susceptibility to illness for both the mother and the newborn, restricting exposure to individuals and everyday activities [11].

People are happier in their well-supportive social network and close relationships and feel more supported by others when they feel loved and wanted in various aspects of their lives and can get help when needed [13].

In India, mothers are provided with customary family and social assistance after giving birth to adjust to their new position [11]. Unfortunately, the need to survive the economic downturn has recently caused this custom to deteriorate. Everyone is busy, even retired grandparents, but still energetically engaged in one work or another to make ends meet. As a result, their help is either limited or non-existent, placing the burden of household duties and baby care on new mothers [7].

A typical mental health issue women go through during pregnancy and the postpartum period is anxiety [14]. Yet though anxiety frequently coexists with depression [15], researchers and medical practitioners have paid little attention [14]. The prevalence of self-reported postnatal anxiety symptoms was 17.8 % at four weeks postpartum, 14.9 % at the third month postpartum, and 15.0 % at the sixth month worldwide [14]. Surveys on postpartum anxiety have found estimates of incidence ranging from 3 % to 43 %, and there is data that it may happen more frequently and independently than postpartum depression (PPD) [16]. Literature implies that a woman is healed from childbirth at that point since they state that the physical healing of reproductive organs after childbirth takes about six weeks [2], [17]. However, the six weeks specified in medical textbooks [18], [19], [20] raises the question of whether this time frame is adequate for all types of deliveries to return to their pre-pregnancy states. Hence, the study aims to determine postnatal women's functional status, social support, and anxiety levels.

## 2 Materials and methods

### 2.1 Study design

A descriptive cross-sectional research design assessed functional status, social support, and anxiety after childbirth.

### 2.2 Setting and sample

The study was conducted in two immunization clinic centers in Bhubaneswar, Odisha. Recruitment took place from 7th November to 24th December 2022. Inclusion criteria include all postnatal women presenting to the immunization clinic at six weeks postpartum, with a live child in the present delivery who can read and write Odia/English. Exclusion criteria include postnatal women who are severely ill, have a psychiatric disorder, whose baby is admitted to NICU, and are not willing to participate in the study (Fig. 2).

The sample size of 200 was calculated using OpenEpi software, version 3, based on the prevalence of a previous study of functional status [7].

### 2.3 Measures/tools

A demographic questionnaire was developed to collect participant information on maternal age, marital status, educational qualification, occupation, family income, complications during antenatal, intranatal, and postnatal, mode of birth, number of pregnancies, and number of living children through the women's self-report. Functional status was measured using the functional level scale after childbirth (FLSAC) [21], a 20-item scale comprising four dimensions. The scale is scored by summing up all the items; scores ranging from 20 to 80 were assigned, depending on their functional status from 1 = Never, 2 = Sometimes, 3 = Most of the time to 4 = all the time. It was categorized into three levels: 20–40 indicates a low return to functional status, 41–61 is moderate, and 62–80 indicates a high return to functional status. For each domain, the following scores were used; for the household activities domain, 7–14 is termed low, 15–21 is moderate, and 22–28 is high; for social and community domain, 3–6 is low, 7–9 is moderate and 10–12 is high; for infant care 6–12 is low, 13–18 is moderate and 19–24 is high; for personal care 4–8 is termed low, 9–12 is moderate, and 13–16 is high. The test-retest reliability coefficient for the scale was 0.97.

Social support was assessed by a Modified multidimensional scale of perceived social support [22], a 12-item five-point Likert instrument with a score of 1 = strongly disagree to 5 = strongly agree. The total score on the self-report scale ranges between 12 and 60. 12–28 indicate low perceived social support, 29–45 is moderate, and 46–60 is high perceived social support. The reported Cronbach's alpha coefficient of MSPSS was 0.88.

**Fig. 1:**
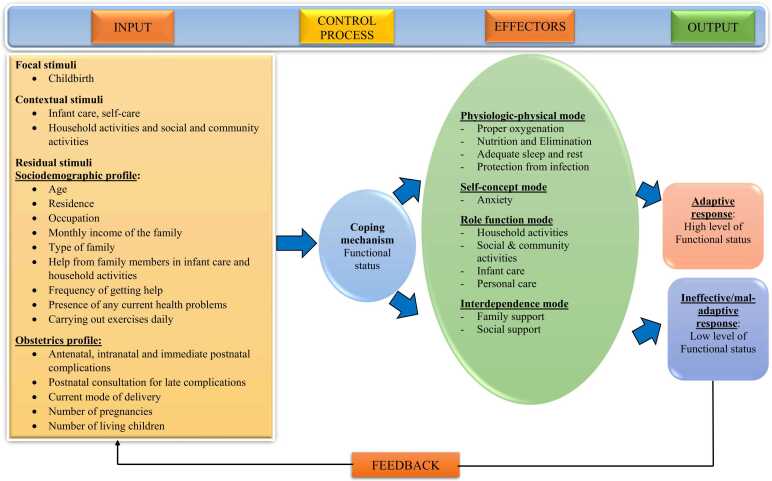
Conceptual framework based on Roy's Adaptation Model.

The current anxiety was measured using the postnatal anxiety scale [16], which is an 18-item four-point Likert scale, and a score ranging from 1 = Not at all, 2 = Sometimes, 3 = Often, and 4 = Almost always was used to collect data. The total score ranges from 18 to 72. The total score is categorized into four categories: 18–31 indicate no anxiety, 32–45 is mild anxiety, 46–59 is moderate anxiety, and 60–72 indicates severe anxiety. The test-retest coefficient for the total scale was 0.98. The conceptual framework is based on Roy's adaptaion model (Fig. 1).

**Fig. 2:**
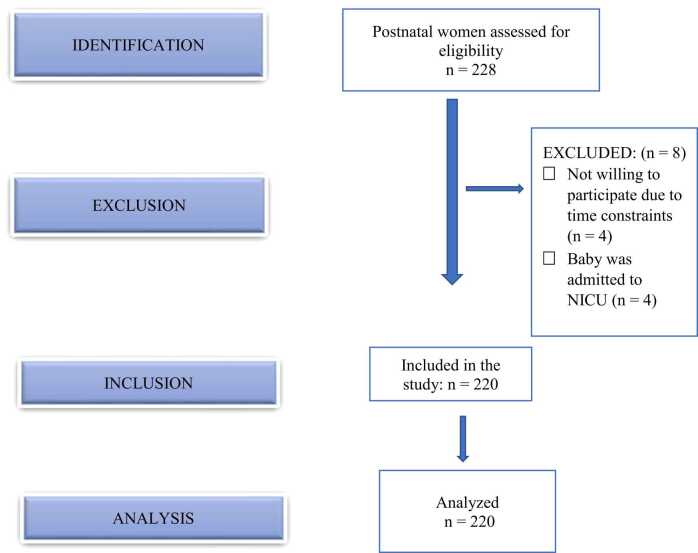
Flow diagram of the study participants as per STROBE guidelines.

**Fig. 3:**
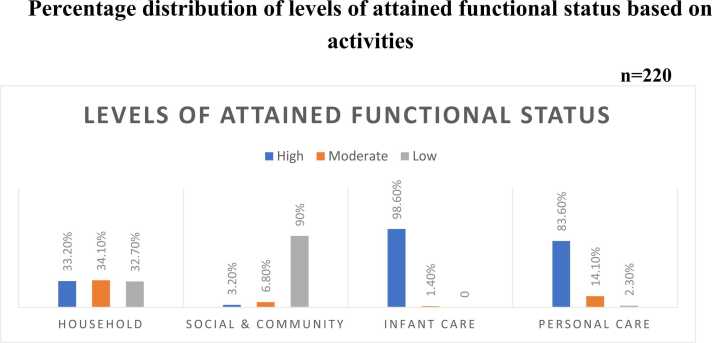
Bar graph showing levels of attained functional status based on activities.

### 2.4 Data collection

When women with their babies visit the immunization clinics, they are made comfortable by asking general questions about the baby and her health. Each postnatal participant was separated in an area in clinics separated with curtains to maintain the participant's privacy. The researcher introduced herself, and the purpose of the study and the total duration to complete the questionnaires were well explained to the woman. The participant information sheet containing information on voluntary participation in the survey, refusal, or withdrawal by the participant at any time without any negative consequence in future care in the hospital was explained by the researcher beforehand. Only those participants who could spare sufficient time were recruited by consecutive sampling technique. Written consent was taken by pen and paper method. Thumbprints were obtained on the consent form for subjects without formal education. Occasionally, when babies were crying, they were made comfortable by assisting the mother with feeding in an area in the clinic, separated by curtains; once the baby was calm, the data-gathering process was resumed. The average duration to complete all questionnaires by one participant was 15–20 min; the researcher reviewed the collected forms for completeness. If any unfilled items were found, the participants were approached immediately to complete those items. The researcher thanked the participant for cooperating and provided health education regarding post-immunization care and breastfeeding. The researcher catered to the non-response by proceeding to another postnatal woman until the required sample size was achieved. An average of four to six participants each day participated in this study.

### 2.5 Ethical consideration

Ethical approval was obtained from the Institutional Ethical Committee vide reference no. IEC/AIIMS BBSR/Nursing/2021-22/32 dated 14.03.2022.

### 2.6 Data analysis

SPSS Software IBM 22.0 (trial version) was used for statistical analysis. Descriptive statistics such as frequency percentage, mean, and standard were used to describe the sociodemographic characteristics of postnatal women. Frequency and percentage were used to describe the level of functional status, social support, and anxiety.

## 3 Results

### 3.1 Recruitment

A total of 228 women visited the immunization clinic during the data collection period, out of whom, women whose children were admitted to NICU and who refused to participate due to time constraints, were excluded. A sample of 220 was enrolled in the study.

### 3.2 Demographic characteristics of the participants

Over half of the women (55%) were between 18 and 28 years old, with a mean and standard deviation of 28 ± 4.1. Two-thirds of the women (65 %) were graduates, nearly half of the women (53.2 %) belonged to urban areas, almost half of the participants (53.6 %) belonged to joint families, the majority (80 %) of women were homemakers, less than one-fifth of the women (43.6 %) had their family income between Rs. 10,001–30,00/- per month, all the study participants got help from a family member while performing infant care and household chores, a majority (91.4 %) of them got help always, more than half of the participants (52.7 %) reported no health issues and a majority (95.9 %) of women did not involve in physical activity. A majority (63.6 %) of participants were primigravida. One-third of the study participants (65.9 %) had one living child. Nearly one-third (64.4 %) of women had no complications during pregnancy. A majority of women (95 %) who underwent vaginal delivery did not have any complications during labor, and (98 %) of all women had no complications during the immediate postnatal period. The only complication reported in the immediate postnatal period was postpartum hemorrhage (2 %). Nearly one-third of women (63.2 %) had a cesarean delivery; out of them, 63.3 % (88/139) were emergency and 36.7 % (51/139) were elective (Table 1).

**Table 1:** Sociodemographic characteristics of postnatal women (n = 220).

Variables	f/Mean ± SD	%
**Age (in years)**	28 ± 4.1	
**Residence**		
Urban Rural	117 103	53.2 46.8
**Education of women**		
No formal education Primary Education Secondary Education Graduation or above	3 13 61 143	1.4 5.9 27.7 65.0
**Occupation of women**		
NIL Govt. Job Private job Self-Employed	176 24 19 1	80.0 10.9 8.6 0.5
**Total monthly family income (in rupees)**		
Less than 10,000 10,001–30,000 30,001–50,000 > 50,000	19 96 45 60	8.6 43.6 20.5 27.3
**Type of Family**		
Nuclear Family Joint Family	102 118	46.4 53.6
**Source of help for household chores**		
Husband Mother Sister Mother-in-law Other family members	15 58 22 96 29	6.8 26.4 10.0 43.6 13.2
**Frequency**		
Rarely Sometimes Always	6 13 201	2.7 5.9 91.4
**Current health issues**		
Yes No	104 116	47.3 52.7
**Daily exercises**		
Yes No	9 211	4.1 95.9
**Complications during pregnancy**		
Yes No	78 142	35.6 64.4
**Complications during labor**		
Yes No	4 216	1.8 98.2
**Complications during immediate postnatal period**		
Yes No	4 216	1.8 98.2
**The current mode of delivery**		
Normal vaginal Cesarean section	81 139	36.8 63.2
Number of living children		
1 ≥ 2	145 75	65.9 34.1

### 3.3 Levels of functional status, social support and anxiety

In this study, more than half of women (59.5 %) had a moderate level of functional status, nearly two-thirds of women (70 %) had a high level of perceived social support, and the majority (87.7 %) of women reported they had no anxiety at six weeks of the postpartum period (Table 2).

**Table 2:** Levels of functional status, social support, and anxiety in postnatal women (n = 220).

Variables	Levels	Range	f (%)
**Functional status**		**20–80**	
	High Level	(62–80)	82 (37.3 %)
	Moderate Level	(41–61)	131 (59.5 %)
	Low Level	(20–40)	7 (3.2 %)
**Social support**		**12–60**	
	High support	(46–60)	154 (70 %)
	Moderate support	(29–45)	65 (29.5 %)
	Low support	(12–32)	1 (0.5 %)
**Anxiety**		**18–72**	
	No anxiety	(18–31)	193 (87.7 %)
	Mild anxiety	(32–45)	25 (11.4 %)
	Moderate anxiety	(46–59)	2 (0.9 %)
	Severe anxiety	(60–72)	-

### 3.4 Comparison of functional status with the mode of delivery

There is a significant difference in functional status with the mode of delivery (*P* = 0.002) (Table 3).

**Table 3:** Comparison of functional status with mode of delivery (n = 220).

Variables		Mode of delivery		*df*	Fisher’s exact value	*P* value
		Normal delivery (n_1_ = 81) f (%)	Cesarean delivery (n_2_ = 139) f (%)			
**Functional status**	High	41 (50.0 %)	41 (50.0 %)	2	12.3	0.002*
	Moderate	40 (30.5 %)	91 (69.5 %)			
	Low	-	7 (100 %)			

Fisher’s exact test, * P value < 0.01 is significant

Table 3 shows a statistically significant difference in functional status with mode of delivery (*P* = 0.002).

## 4 Discussion

The Main finding of this study showed that most (59.5 %) women returned to a moderately functional status six weeks after giving birth. In particular, women achieved high levels of child care (98.6 %) and personal care (83.6 %), moderate levels of resumption to household tasks (34.1 %), and low levels of resumption to social and communal activities (90 %). Also, most women (70 %) had high perceived social support and no anxiety (87.7 %) after six weeks of childbirth (Fig. 3). In this study, functional status was statistically significant with vaginal delivery in the current pregnancy.

Our study showed a high level of perceived social support from family members among postnatal women at six weeks postpartum. In India, living with extended family is expected. The partner acts as the principal supporter for the mother when other family members are not present with the mother [10], [12], [23], [24], followed by grandparents, who also prove to be an essential support person [25]. The support culture prevails among immigrant Indian women as neighbors and other people, such as landladies, provided assistance by lodging with the women or paying frequent visits to those without extended family members residing in their homes [23]. Caucasian women in the USA have expressed that they have support networks and partners to turn to. Also, these women spoke highly of the assistance of the visiting nurses who regularly visited them following the delivery [26]. Most Turkish women receive assistance from close family and friends for domestic chores and baby care after giving birth as part of their cultural tradition [27].

A woman experiences numerous biological, psychological, and social changes throughout pregnancy. A mother often feels stressed just after giving birth due to her exhaustion after the delivery and needing to take on new tasks immediately [28].

Present study findings showed that most women had no anxiety after six weeks postpartum, probably due to high perceived social support. A study conducted in India found that it is usual for a woman to experience altered or melancholy moods during the third trimester and after delivery [29]. Among Indian communities in Australia, the family was looked to for support in addressing changing moods. Therefore, mothers' feelings of sadness were most frequently viewed as a natural reaction to giving birth and were not classified as illnesses, diseases, or conditions needing medical or psychological treatment [11]. In our study, where nearly half of the women live in joint families, social support meant fewer family and home life pressures. Most women were graduates, so they felt comfortable sharing their worries when they went for a routine pregnancy checkup, which likely alleviated their fears and anxieties following delivery. This finding is consistent with another study conducted in South India [30].

The current study reported that functional status was statistically significant with mode of delivery. The fact that vaginal birth recipients resume functional ability earlier than cesarean section recipients is an expected outcome [2], [31]. This finding contradicts an earlier study conducted in Iran, which shows no significant relationship between the type of delivery and functional status [5]. Among Turkish women, the mode of delivery did not affect functional status [1]. There needs to be more research done in this area to understand how delivery mode affects functional status.

Based on the present study findings, it was evident that functional status was not associated with social support. This finding is consistent with the study findings of Australia [32] and Turkey [31] where social support and the rate of postpartum recovery to a fully functional state were not correlated. Contrary to our study in Taiwan, social support is a crucial factor in successfully adjusting to a new mother's role [33]. A woman who receives great social support during the postpartum period feels less fatigued, has more time for herself, spends more time with the baby, has a more robust postpartum functional condition, and finds it easier to adapt to her physical and psychological needs as well as to motherhood [34]. A meta-synthesis revealed that health professionals' advice on baby growth, hygiene, vaccines, breastfeeding, nutrition, and practical parenting advice is valued by women and impacts their ability to function normally after giving birth [35]. Given that receiving assistance predicts maternal functioning, nursing care should incorporate social support intervention to enhance maternal functioning [4].

The present study also found an association between functional status and anxiety at six weeks of childbirth, concordant with a study conducted in New Jersey [6]. Another study in Australia found a significant inverse relationship between anxiety and functional status [32], [36]. A study conducted in Pennsylvania found that women who tested positive in state anxiety and depression screens resulted in poorer breastfeeding outcomes [37]. The present study does not address a cause-and-effect relationship between these parameters because of the cross-sectional nature of our research; however, FLSAC scores for mothers with no or mild anxiety are higher.

In a previous study [33] for postpartum women, greater perceived social support is associated with less postpartum anxiety, but our study failed to support that. Studies conducted in Australia [32], [38] show that having a supportive social network during the postnatal period reduces anxiety and a stress-reduction strategy, lowering psychological discomfort, which positively impacts physical health [38]. In California, consistent social support can help prevent the postpartum manifestation of perinatal mood and anxiety disorders [39]. Another study conducted in Turkey found that support nearly disappears as the weeks go on, leaving the mother and child alone [26], [27]. The support group typically assumes that a new mother can care for her child and her family independently. Due to these cultural trends, there is a greater anxiety score in the later postpartum weeks [25]. In Nepal, anxiety was significantly decreased by positive relationships with spouses and mothers-in-law and by their encouragement of primiparous mothers [15].

### 4.1 Study limitations and strengths

The present study was conducted in two hospitals representing the population having access to healthcare facilities. A community with a door-to-door approach would have bettered the generalizability of the study with representation from urban, rural, and tribal women. However, it was not feasible due to time constraints. The FLSAC scale was developed to evaluate the functional status of women. It assesses the woman's readiness to take on her responsibilities before giving birth, including assuming infant care. However, the scale does not evaluate women's perception of such roles. Women's postpartum recovery periods differ depending on the situation; we could not assess functional status over time due to time constraints. We did not analyze emergency/elective cases separately to evaluate their effect on functional status. Prenatal psychological status was not evaluated in our study; as a result, pre-existing psychological disorders may be misinterpreted as postpartum anxiety. We cannot be sure whether it was pre-existing or was a fresh episode of anxiety. In our study, mothers were asked to recall their experiences post-birth; hence, their memories were prone to recall bias. Courtesy and social desirability bias might have been there, as the researcher was in her uniform.

To the researcher's best knowledge, this was the first study assessing functional status, social support, and anxiety among postnatal women of eastern India. The descriptive study design gave scope for clarification of doubt and a good interpersonal relationship with low attrition.

### 4.2 Recommendations

Nurses and midwives must understand the concept of functional status after childbirth and the importance of family, particularly women's husbands and mothers, as providers of social support in the postnatal period. Given that women's postpartum functional capacity varies, postpartum follow-ups should be done for a more extended period so that actual health problems faced by the mother can be explored and addressed. Policies need to be formulated and communicated to the stakeholders to assess the mental health status of postnatal women immediately after delivery and during postnatal hospital visits. Studies with a mixed methodology approach may be undertaken to explore in-depth perceptions and experiences of women regarding attainment of functional status, received or perceived social support, and anxiety during the postpartum period. Studies should be carried out in culturally diverse settings with a large sample size.

## 5 Conclusion

In concordant with many prior studies, the six weeks following birth should be reconsidered; even while physiological recovery occurs within six weeks of delivery, much more time was required to adjust for the ability to adapt to parental and social roles. During pregnancy, women should be sensitized about the maternal role changes and physiological changes after childbirth through nurses and midwives. They should also educate and encourage family members and partners to provide support during postpartum to cope with crises.

## Ethical Issues

None to be declared.

## Source of Financial Support

Nil.

## Declaration of Competing Interest

The authors declare that they have no known competing financial interests or personal relationships that could have appeared to influence the work reported in this article

## Data Availability

The datasets generated during and analyzed during the current study are available from the corresponding author upon reasonable request.
